# The association between work capacity, quality of life, and organizational climate among nurses

**DOI:** 10.47626/1679-4435-2025-1373

**Published:** 2025-07-08

**Authors:** Isabela Saura Sartoreto Mallagoli, Aline Bicalho Matias, Evandro Piccinelli da Silva, Maria Aline do Nascimento Oliveira, Aristeia Nunes Sampaio, Angélica Gonçalves Silva Belasco

**Affiliations:** 1 Universidade Federal de São Paulo, São Paulo, SP, Brazil; 2 Universidade Federal do Acre, Rio Branco, AC, Brazil

**Keywords:** nursing, quality of life, health personnel, occupational health., enfermagem, qualidade de vida, pessoal de saúde, saúde ocupacional.

## Abstract

**Introduction:**

Work capacity is the ability to perform one’s job both currently and in the future.

**Objectives:**

We investigated the association among factors related to work capacity, quality of life, organizational climate, sociodemographics, and work activities among nurses at a university hospital.

**Methods:**

This cross-sectional study included 280 nurses from a tertiary referral hospital in São Paulo, Brazil. The Work Capacity Index, the Escala de Clima Organizacional, the 36-item Short-Form Health Survey, and a sociodemographic questionnaire were applied.

**Results:**

Work capacity was moderate-to-low in 45.7% of participants. In the multiple linear regression analysis, it was lower with increasing age (p = 0.019), musculoskeletal disease (p < 0.001), cardiovascular disease (p = 0.001), and functional limitations (p < 0.001) and higher with perceived support from management and the organization (p = 0.017) and with better quality of life scores in the physical aspects (p < 0.001), general health (p = 0.001), and mental health (p < 0.001) domains.

**Conclusions:**

Significant direct correlations were found between work capacity and support from management and the organization, physical aspects, general health, and mental health. Significant inverse correlations were found between work capacity and older age, musculoskeletal disease, cardiovascular disease, and functional limitations.

## Abstract (pt)

**Introdução:**

A capacidade para o trabalho refere-se a quão bem e capaz o trabalhador está, ou estará, para realizar suas atividades laborais.

**Objetivos:**

Avaliar a associação entre a capacidade para o trabalho e os fatores qualidade de vida e clima organizacional, além de aspectos sociodemográficos e laborais, de profissionais de enfermagem de um hospital universitário.

**Métodos:**

Estudo transversal realizado com 280 trabalhadores de enfermagem de um hospital público de referência em alta complexidade, localizado em São Paulo, Brasil. Foram utilizados os instrumentos Índice de Capacidade para o Trabalho, Escala de Clima Organizacional e 36-item Short-Form Health Survey e um questionário de caracterização.

**Resultados:**

A capacidade para o trabalho foi considerada inadequada em 45,7% dos participantes. Na análise de regressão linear múltipla, a capacidade diminuiu com o aumento da idade (p = 0,019), presença de doenças musculoesqueléticas (p < 0,001) e cardiovasculares (p = 0,001) e restrição funcional (p < 0,001). Por outro lado, aumentou quando o indivíduo percebia apoio da chefia e da organização (p = 0,017) e quando tinha melhores escores de qualidade de vida nos domínios aspectos físicos (p < 0,001), estado geral (p = 0,001) e saúde mental (p < 0,001).

**Conclusões:**

Apoio da chefia e da organização, aspectos físicos e estado geral de saúde e saúde mental apresentaram associação significativa positiva com a capacidade para o trabalho, enquanto idade mais avançada, presença de doenças musculoesqueléticas e cardiovasculares e restrição funcional apresentaram associação significativa inversa.

## INTRODUCTION

Nurses work in many areas of health care, each with its own peculiarities and challenges, but they share a daily routine involving occupational risks. Such risks include physiological and psychosocial factors, as well as physical, mental, and social exhaustion. In a routine replete with stressors, these professionals are subject to potential health problems and physiological and mental imbalance, which can damage or worsen health conditions and compromise work capacity.^1,2^

Work capacity is related to the demands of work, health, and mental resources. It has been defined as how well workers are now, how well they will be in the near future, and how capable they are of performing their tasks.^3^ Low work capacity has been associated with negative outcomes, including physical and mental illness, sick leave, job dissatisfaction, productivity loss, increased intention to leave the profession, lower employability, unemployment, career abandonment, early retirement, disability retirement, and death.^4-6^

Sociodemographic factors, physical and mental health, and working conditions can be considered determinants of work capacity.^2^ Organizational climate, which stands out among work conditions, is related to the values workers attribute to the practices and behaviors of their work context. Organizational climate is multidimensional, temporary, and regulatory, guiding human behavior within the organization.^7,8^ It has been described as the shared perception of the work environment.^7^

Another important aspect of work capacity is quality of life (QoL), defined as an individual’s perception of his or her position in life within the cultural context and value systems in which he or she lives and in relation to his or her goals, expectations, standards and concerns. QoL incorporates physical health, psychological state, independence level, social relationships, personal beliefs, and their relationships with environmental characteristics. Thus, it reflects what individuals feel in relation to their cultural, social, and environmental context.^9^

Constructs related to organizational climate, QoL, and work capacity intersect in the daily life of nurses.^2,10^ Although studies on work capacity have sometimes investigated it in association with QoL or organizational climate, there is a need to assess them together in light of variables previously described as fundamental for work capacity assessment, such as age, gender, remuneration, workload, and type of work. Thus, the broader innovative analysis of the present study can help advance knowledge on the association between work capacity, QoL, and organizational climate, sociodemographic aspects, and work characteristics among nurses.

## METHODS

This cross-sectional^11^ study was conducted between May 2021 and February 2022 at a public university hospital in São Paulo, SP, Brazil that is a tertiary referral center for highly complex cases. The sample included nursing workers (registered nurses, nurse technicians, and licensed practical nurses) from different hospital sectors and outpatient areas who had worked at the hospital for more than 1 year and who were not on leave or vacation.

Based on a total population of 982 nursing workers at the hospital, the sample size was calculated using the most conservative value for the tested proportion (50%), with a sampling error of 5% and a confidence level of 95%,^12^ resulting in a minimum of 277 participants. A total of 280 responses were obtained.

Using the RANDBETWEEN function in Microsoft Excel, 471 people were randomly selected and invited to participate in the study by telephone and/or email. Of these, 280 agreed, a response rate of 59.4%. This response rate was higher than other studies that collected data via telephone or email (< 15%).^13,14^ After accepting and digitally signing an informed consent form, the evaluation questionnaires were sent via Google Forms. The responses were automatically uploaded to a database, thus avoiding typing errors. After the sociodemographic questionnaire was completed, the other instruments were applied in random order to avoid bias.

Individual, work, and health data were collected using a sociodemographic questionnaire developed specifically for this purpose. Work capacity was measured using the Work Capacity Index, which has satisfactory psychometric properties and good performance in terms of construct validity, criterion validity, and reliability, with a Cronbach’s alpha of 0.80 in nurses. The questionnaire assesses 7 dimensions: current work capacity and current capacity compared with the worker’s previous best; work capacity in relation to work demands; current number of illnesses diagnosed by a doctor; estimated loss of work capacity due to illnesses; absences from work due to illness; work capacity prognosis; and mental resources. Based on the final score, work capacity is classified as low (7 to 27 points), moderate (28 to 36), good (37 to 43) or excellent (44 to 49).^3,15^

To assess organizational climate, we used the Escala de Clima Organizacional (Organizational Climate Scale - ECO), which was developed and validated in Brazil, with internal consistency between 0.78 and 0.92. The scale analyzes 5 factors: 1) “Support from management and organization” through 21 items on emotional, structural, and operational support behavior by management and the organization regarding daily work activities; 2) “Reward” through 13 items on the various ways the company rewards quality, productivity, effort, and employee performance; 3) “Physical comfort” through 13 items on the physical environment, safety, and comfort the company provides to workers; 4) “Control/pressure” through 9 items on the control and pressure the company and supervisors exert on worker behavior and performance; and 5) “Coworker cohesion” through 7 items on teamwork and the bond between coworkers. For factors 1, 2, 3 and 5, higher scores indicate a better organizational climate, with values > 4 considered good, and values < 2.9 considered poor. The opposite is the case for factor 4 (control/pressure), with higher scores indicating a poorer organizational climate. Thus, values > 4 indicate a poor climate and values < 2.9 indicate a good climate.^8^

The 36-item Short-Form Health Survey (SF-36), which has been translated, validated, and culturally adapted for Brazil, was used to assess health-related QoL. The items of this generic health questionnaire are grouped into 8 dimensions: functional capacity, physical aspects, emotional aspects, pain, general health status, vitality, social aspects, and mental health. Scores range from 0 to 100, with 0 being the worst state and 100 being the best state of QoL for each dimension^16^. In the present study, scores were dichotomized as < 70 for poor QoL and ≥ 70 for good QoL.^17^

Descriptive and inferential analyses of the sample were performed in jamovi. The Spearman correlation test was applied to assess the relationship between the variables of interest. The data then underwent bivariate analysis with the chi-square or Fisher’s exact tests for categorical variables and analysis of variance or the Kruskal-Wallis test for numerical variables. For multivariate analysis, multiple linear regression was used with backward modeling, assessing the coefficient of determination (R^2^ and adjusted R^2^), autocorrelation, multicollinearity, the normality test for residuals, and analysis of variance. Only statistically significant variables (p ≤ 0.05) were maintained in the final model.

The study was approved by the 38952820.2.0000.5505 Research Ethics Committee, through Plataforma Brasil (no. 4.559.038). The participants provided written informed consent after being informed about the study’s methodology, guaranteed data confidentiality, voluntary participation, and the right to withdraw at any time, including withdrawal of their data.

## RESULTS

The sample consisted of 280 nursing workers, who were generally in 40-59-year age range (43.9%), married (57.5%), female (84.3%), and had a graduate education level (47.1%). The majority provided direct care to patients (84.3%) and worked in the intensive care unit, surgical center, materials center, or the emergency department (40.36%). More than half (53.2%) rated their physical health as average or poor, and nearly half rated their mental health as poor (49.3%). Occupational illnesses were reported by 49.6%, and 97 workers (34.6%) reported spending more than 2 hours commuting to work.

The organizational climate was assessed as fair or poor for almost all factors, except for the “control/pressure” factor, which was good. Functional capacity was the only dimension of QoL to score above the cutoff line (74.1), with vitality having the lowest score (54.1). Work capacity was moderate-to-low in 45.7% of workers (Table 1).

**Table 1:** Distribution of sociodemographic and work variables according to variable type and bivariate analysis with work capacity, São Paulo, SP, Brazil, 2022

Variable/category	n(%) or n ± SD	p-value
Age range (years)		
≤ 39	76 (27.2)	0.110
40-49	123 (43.9)	
50-59	60 (21.4)	
≥ 60	21 (7.5)	
Sex		
Female	236 (84.3)	0.519
Male	44 (15.7)	
Marital status		
Married	161 (57.5)	0.342
Single	69 (24.6)	
Separated/divorced/widowed	50 (17.9)	
Education level		
High school	75 (26.8)	0.370
Undergraduate	73 (26.1)	
Graduate	132 (47.1)	
Self-assessment of physical health		
Good	131 (46.8)	< 0.001
Average	122 (43.6)	
Poor	27 (9.6)	
Self-assessment of mental health		
Good	142 (50.7)	< 0.001
Average	108 (38.6)	
Poor	30 (10.7)	
Occupational illness ^*^	139 (49.6)	< 0.001
Musculoskeletal diseases ^*^	129 (46.0)	< 0.001
Cardiovascular diseases^*^	41 (14.6)	0.002
Emotional disorders^*^	35 (12.5)	0.030
Endocrine or metabolic diseases^*^	44 (15.7)	0.010
Other diseases ^*^	44 (15.7)	0.005
Functional limitation^*^	50 (17.8)	< 0.001
Patient contact type		
Direct/direct assistance	236 (84.3)	0.142
Indirect/administrative function	13 (4.6)	
Both	31 (11.1)	
Commute to work (hours)		
≤ 1	43 (15.3)	0.022
1.1-2	97 (34.6)	
2.1-3	74 (26.4)	
≥ 3.1	66 (23.6)	
Weekly work hours	41.5 ± 10.7	0.044
Organizational climate		
Support from management and the organization	3 ± 0.7	0.033
Reward	2.1 ± 0.6	0.084
Physical comfort	2.8 ± 0.7	0.278
Control/pressure	2.7 ± 0.6	0.041
Coworker cohesion	3.6 ± 0.7	0.028
Quality of life		
Functional capacity	74.1 ± 21.9	< 0.001
Physical aspects	69.5 ± 37.1	< 0.001
Pain	59.8 ± 23.3	< 0.001
General condition	68.3 ± 18.0	< 0.001
Vitality	54.1 ± 22.9	< 0.001
Social aspects	66.5 ± 26.7	< 0.001
Emotional aspects	66.8 ± 39.9	< 0.001
Mental health	65.1 ± 20.8	< 0.001
Work capacity		
Excellent	26 (9.3)	-
High	126 (45.0)	
Average	112 (40.0)	
Low	16 (5.7)	
Total	280 (100.00)	

P-values refer to the chi-square test, Fisher’s exact test, analysis of variance, or the Kruskal-Wallis test.

% = relative frequency; SD = standard deviation; M = mean; N = absolute frequency.

* Situation or diseases reported.

There was a direct positive correlation between work capacity and each dimension of the ECO and SF-36, except “control/pressure” (p = 0.52)(Table 2).

**Table 2:** Correlation between work capacity and factors related to organizational climate and quality of life, São Paulo, SP, Brazil, 2022

Factor	Spearman correlation	p-value
Organizational climate		
Support from management and the organization	0.268	< 0.001
Reward	0.185	0.002
Physical comfort	0.260	< 0.001
Control/pressure	-0.038	0.526
Coworker cohesion	0.233	< 0.001
Quality of life		
Functional capacity	0.526	< 0.001
Physical aspects	0.443	< 0.001
Pain	0.565	< 0.001
General condition	0.567	< 0.001
Vitality	0.532	< 0.001
Social aspects	0.431	< 0.001
Mental health	0.483	< 0.001

Table 3 presents an association model for the Work Capacity Index, with work capacity scores as the dependent variable in relation to age/health status and factors from the ECO and SF-36. This final model met all validity assumptions (R^2^ 0.55; adjusted R^2^ 0.54; lack of multicollinearity [p > 0.05]); all variance inflation factors < 5; and a p-value > 0.05 in the normality test of the residuals. The p-value in analysis of variance was < 0.001. The results indicate that increasing age, musculoskeletal and cardiovascular disease, and functional limitations were associated with lower work capacity. However, better perceived support from management and the organization and better scores for physical aspects, general health, and mental health were associated with higher work capacity.

**Table 3:** Multiple linear regression analysis between Work Ability Index and associated variables, São Paulo, SP, Brazil, 2022

Variable		95%CI		p-value
	Estimates	Lower	Upper	
Age	-0.059	-0.108	-0.009	0.019
Musculoskeletal diseases	-2.182	-3.117	-1.248	<0.001
Cardiovascular diseases	-2.093	-3.354	-0.833	0.001
Reported functional limitation	-2.255	-3.438	-1.072	<0.001
Organizational Climate Scale				
Support from management and organization	0.782	0.140	1.424	0.017
SF-36				
Physical aspects	0.027	0.014	0.040	<0.001
General condition	0.051	0.020	0.082	0.001
Mental health	0.077	0.053	0.102	<0.001

SF-36 = 36-item Short-Form Health Survey.

## DISCUSSION

Our sample is comparable to that of other studies in similar populations.^2,4,18,19^ The results showed that age, disease, functional limitations and factors related to organizational climate and QoL were associated with work capacity. The observed frequency of low or moderate work capacity (45.7%) was higher than that found by other studies.^2,20^ Notably, in a systematic review and meta-analysis of 24,728 nursing workers from 14 countries, the prevalence of moderate or low work capacity was 26.8% (95%CI: 22.4%-31.5%).^18^ These data indicate a higher level of impaired work capacity in our sample, reinforcing the need for assessment and monitoring to improve the related variables.

According to the literature, work capacity is an important indicator among nurses, useful for health monitoring and early interventions since it covers the physical, psychological and functional dimensions, as well as professional skills, organization and working conditions, worker engagement, and constructed identity. Improving these factors can lead to better QoL standards, especially considering the relationship between work and population aging.^2,21^ Seeking to improve the work capacity of nurses can benefit the worker, the company, and the general public, who are treated by nurses at different points throughout life.

Numerically speaking, nurses are the largest category of health care workers in Brazil and the world. They are exposed to occupational risk, stress, and high work demands on a daily basis. It is thus essential to invest in initiatives to promote, maintain, and care for their work capacity, including programs aimed at physical and mental health, encouraging self-care, improving the work environment, and providing staff rest areas, which can help them perform their duties in greater safety, resulting in improved care quality.^2,21^

Age was negatively associated with work capacity, indicating that it is a fundamental variable, since aging remodels one’s physical, social, and psychological resources.^2,18^ Research on work capacity is generally motivated by population aging and the need to extend the careers of employees. It seeks to understand how people can work for so many years and to what extent it is possible to maintain work capacity, considering factors such as the content and demands of work, changes in professional life, advances in technology, globalization, and the growing demand for quality and increased productivity.^22^

As in other studies of nurses, musculoskeletal and cardiovascular diseases and functional limitations were negatively associated with work capacity in our sample. One obstacle to overcoming health problems is returning to work without adequate rehabilitation support. Continuing to work in the same sector or remaining exposed to the situations that caused the illness reduces work capacity.^20,23^

Thus, occupational health services must monitor and guide professionals who return from medical leave with some type of functional limitation in order to adapt, in association with management, their work activities to their health restrictions. This adaptation must allow workers to continue performing their duties in a manner compatible with their new functional level, which may be temporary or permanent.^20,23,24^

Recognizing the health needs of workers is an important factor in mobilizing them to minimize the risk of occupational illness. Promoting healthy work environments and investing in health education can improve self-care and reduce vulnerability among workers.^24-26^

In addition to these determinants, we assessed factors related to organizational climate. As indicated in the literature, our sample’s scores reflected an average-to-poor perception of this construct.^7,10,27^ Understanding how nursing workers perceive their context and behave in organizations involves consideration of both the positive and negative effects of the work environment, as well as their influence on satisfaction, well-being, and care quality.^7,27^

The correlation analysis indicated that organizational climate influenced work capacity in the following factors: support from management and the organization, reward, physical comfort, and coworker cohesion. In the linear regression analysis, support from management and the organization remained associated with work capacity, possibly because it reflects affective, structural, and operational support behaviors towards employees in their daily work activities.^8^

Interaction between workers and managers, such as interpersonal conflicts, collaboration, and worker participation in changes, can be prioritized by the institution in an effort to improve the organizational climate and, consequently, work capacity and QoL.

Several studies have demonstrated an association between work capacity and factors in organizational climate, such as: support from management and the organization, psychosocial and work environment conditions, limited autonomy, high levels of responsibility, excessive mental and physical workload, interpersonal conflict with managers, understaffing and a lack of materials, rewards, limited professional development, high control/pressure from management, lack of coworker cohesion, and unsatisfactory interpersonal relationships.^1,4,7,20^ These associations reinforce the importance of organizational climate for nurses by highlighting how they are affected by working conditions, relationships with colleagues, the company, and management, interpersonal communication, and rewards and benefits. They also reveal statistically significant associations between dimensions of organizational climate and factors such as job satisfaction, motivation to work, professional turnover, etc.^7,27^

Organizational support and organizational climate can be considered protective factors, provided they are perceived positively by workers. Organizations tend to promote the well-being of workers when they demonstrate appreciation and recognition through adequate remuneration and working conditions, benefits and interactional support. Likewise, when workers receive support from the organization, they generally seek to maintain a pleasant work environment, resulting in better productivity and perceived QoL.^7,27^

The mean QoL scores in this study were lower than those of university hospital nurses in Italy, who scored 87.36 for functional capacity, 68.78 for physical aspects, 67.59 for pain, 61.42 for general health status, 54.39 for vitality, 61.65 for social aspects, 66.84 for emotional aspects, and 65.11 for mental health.^19^

In our regression analysis, only physical, general health, and mental health dimensions of QoL were significantly associated with work capacity. Most QoL dimension scores in this sample were below 70 points. This is similar to the results of other studies, which also found significant correlations between environment, sleep and rest, work capacity, fatigue, cognitive aspects, and work overload. Other variables that have been shown to affect QoL include understaffing and a lack of materials, inadequate infrastructure, stress, low wages, a disorganized environment, poor interpersonal relationships with colleagues, patients and managers, a lack of social support, a lack of physical activity and leisure, irregular sleep, and a lack of security.^28,29^

Maintaining QoL among nurses requires careful attention, especially due to their high occupational risk and its impact on the care they provide. Although the QoL of nursing team members is a fundamental topic, effective and lasting institutional policies are still lacking.^24,28,29^

Work capacity is an important indicator that can be integrated into monitoring strategies and guide early interventions to improve QoL and the organizational climate in the complex system of nursing work. Hence, our results can be used in initiatives to promote better work environments, strengthen QoL, and increase work capacity.

However, this study’s cross-sectional design is inherently limited, since causal relationships or generalizations cannot be established from its results. In addition, the sample consisted solely of government employees from a single hospital, which also limits the extrapolation of the results to other contexts.

## CONCLUSIONS

Work capacity was moderate or low in 45.7% of the sample. Support from management and the organization, physical aspects, general health status, and mental health were positively associated with work capacity. However, there were significant inverse correlations between work capacity and older age, musculoskeletal or cardiovascular diseases, and functional limitations.
